# Gestational zinc deficiency impairs brain astrogliogenesis in rats through multistep alterations of the JAK/STAT3 signaling pathway

**DOI:** 10.1016/j.redox.2021.102017

**Published:** 2021-05-19

**Authors:** Suangsuda Supasai, Ana M. Adamo, Patricia Mathieu, Regina C. Marino, Adelaide C. Hellmers, Eleonora Cremonini, Patricia I. Oteiza

**Affiliations:** a Department of Nutrition, University of California, One Shields Avenue, Davis, CA, 95616, USA; b Department of Environmental Toxicology, University of California, One Shields Avenue, Davis, CA, 95616, USA; c Department of Molecular Tropical Medicine and Genetics, Faculty of Tropical Medicine, Mahidol University, Bangkok, 10400, Thailand; d Department of Biological Chemistry and IQUIFIB (UBA-CONICET), Facultad de Farmacia y Bioquímica, Universidad de Buenos Aires, Buenos Aires, Argentina

**Keywords:** Zinc, Brain development, STAT3, Tubulin, Astrogliogenesis, Tubulin oxidation

## Abstract

We previously showed that zinc (Zn) deficiency affects the STAT3 signaling pathway in part through redox-regulated mechanisms. Given that STAT3 is central to the process of astrogliogenesis, this study investigated the consequences of maternal marginal Zn deficiency on the developmental timing and key mechanisms of STAT3 activation, and its consequences on astrogliogenesis in the offspring. This work characterized the temporal profile of cortical STAT3 activation from the mid embryonic stage up to young adulthood in the offspring from dams fed a marginal Zn deficient diet (MZD) throughout gestation and until postnatal day (P) 2. All rats were fed a Zn sufficient diet (control) from P2 until P56. Maternal zinc deficiency disrupted cortical STAT3 activation at E19 and P2. This was accompanied by altered activation of JAK2 kinase due to changes in PTP1B phosphatase activity. The underlying mechanisms mediating the adverse impact of a decreased Zn availability on STAT3 activation in the offspring brain include: (i) impaired PTP1B degradation via the ubiquitin/proteasome pathway; (ii) tubulin oxidation, associated decreased interactions with STAT3 and consequent impaired nuclear translocation; and (iii) decreased nuclear STAT3 acetylation. Zn deficiency-associated decreased STAT3 activation adversely impacted astrogliogenesis, leading to a lower astrocyte number in the early postnatal and adult brain cortex. Thus, a decreased availability of Zn during early development can have a major and irreversible adverse effect on astrogliogenesis, in part via multistep alterations in the STAT3 pathway.

## Highlights

• Maternal zinc deficiency disrupts offspring's brain cortical STAT3 activation.
• Impaired PTP1B proteasomal degradation inhibits JAK/STAT3 activation.
• Tubulin oxidation and decreased interactions with STAT3 can affect STAT3's nuclear localization.
• Zinc deficiency impairs STAT3 acetylation.
• Altered STAT3 activation leads to astrogliogenesis disruption which extends into adulthood.

## Graphical abstract

**Figure:**
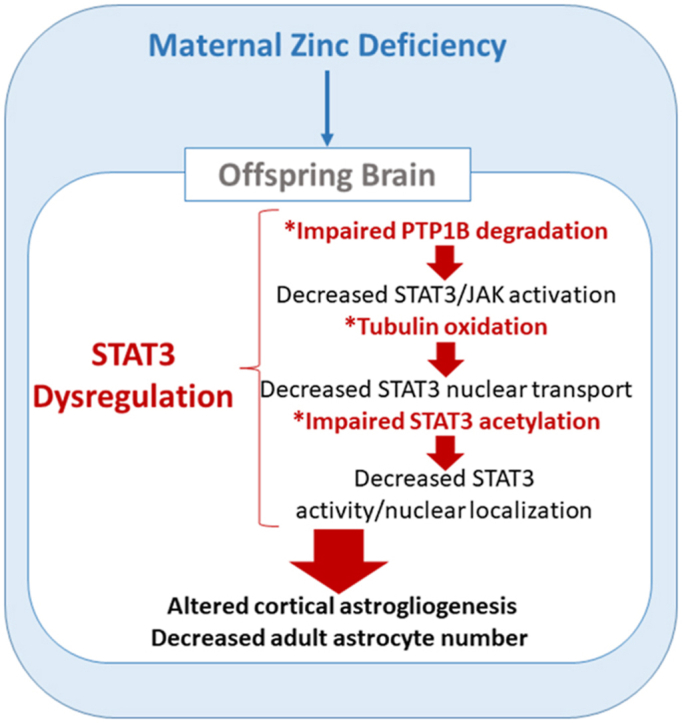

## Abbreviations

BMP: bone morphogenetic protein
C: control (zinc sufficient diet)
CT: cortical tissue
CT-1: cardiotrophin-1
E: embryonic day
bFGF: basic fibroblast growth factor
GAPDH: glyceraldehyde 3-phosphate dehydrogenase
GFAP: glial fibrillary acidic protein
hnRNP: heterogeneous nuclear ribonucleoprotein A1
JAK2: Janus kinase 2
LIF: leukemia inhibitory factor
MZD: marginal zinc diet
NSC: neural stem cells
P: postnatal day
PTP1B: protein-tyrosine phosphatase 1B
SHP2: SH2 containing protein tyrosine phosphatase-2
STAT: signal transducer and activator of transcription
SVZ: subventricular zone
S100β: S100 calcium-binding protein β
VZ: ventricular zone
Zn: zinc

## 1 Introduction

Zinc (Zn) is essential for all living organisms and is one of the most abundant metals in the body. Zn plays a key role in the physiology of the nervous system. In this regard, Zn is a structural or functional component for a large number of proteins including enzymes, receptors, and transcription factors [1,2], regulates cellular redox balance [3], modulates synaptic transmission and intracellular signaling [2], and is necessary for neural stem cell (NSC) proliferation [4,5]. In fact, perturbations of Zn homeostasis are implicated in many neurological and psychiatric disorders [2].

Given the critical role of Zn in the regulation of cell proliferation, differentiation, and survival, a decreased zinc availability during early development can have a major impact on brain structure and function [6]. In this regard, Zn deficiency induces apoptotic death of NSCs, neurons and glia, both *in vivo* and *in vitro* [[5], [6], [7]]. Dietary marginal Zn deficiency imposed throughout gestation decreases NSCs proliferation [5] and affects neurogenesis [4]. The latter results in a lower number of cortical glutamatergic neurons in the young adult brain, even upon postnatal Zn repletion. On the other hand, the impact of Zn deficiency during early development on brain astrogliogenesis is unknown. Given that NSCs not only give rise to neurons but also to oligodendrocytes and astrocytes, it is possible that Zn deficits could also affect astrogliogenesis.

Almost half the cells in the adult human brain are glial cells [8], astrocytes being the most abundant cell type and playing a wide range of key roles in brain development and function [9]. Astrogliogenesis follows neurogenesis during mammalian brain development [10,11]. Rodent corticocerebral astrogliogenesis mainly takes place during the first three postnatal weeks and consists of two concurrent regulatory processes: (1) astrocytic progenitor cell fate decisions and (2) local astrocyte proliferation [12,13]. Studies from *in vitro* and mouse models indicate that bone morphogenetic protein (BMP)-Smads [13,14], Notch [13,15], and Janus kinase 2 (JAK2)/signal transducer and activator of transcription (STAT) signaling pathways control timely astrogliogenesis [16,17].

Our recent study showed that Zn deficiency impairs the activation of STAT1 and STAT3 in the developing rat brain and in neuroblastoma cells in culture, in part through oxidative stress-mediated mechanisms [18]. We observed that Zn deficiency affects the activating STAT1/3 tyrosine phosphorylation, STAT1/3 nuclear translocation, DNA binding and *trans*-activating activity [18]. Thus, alterations in the timely activation of STAT3 could affect astrogliogenesis. However, little is known on how developmental zinc deficiency can affect brain STAT3 activation during the period of active astrocyte generation and if this can have an impact on the adult brain astrocyte number and distribution. The latter could contribute to the development of long-term cognitive deficits and neurological diseases.

Based on the above, this study investigated the effects of a marginal Zn nutrition imposed to rats from gestation day 0 until postnatal day (P) 2, on STAT3 activation, astrogliogenesis, and its consequences on corticocerebral astrocyte population in the young adult brain. The mechanisms of STAT3 activation were characterized at different developmental stages (embryonic day (E) 14, E19, P2, P56). A marginal decrease in fetal Zn availability caused alterations in STAT3 activation and astrogliogenesis leading to a lower number of cortical astrocytes in the young adult brain. These findings stress the relevance of a proper zinc availability during critical developmental periods for normal brain development and function.

## 2 Materials and methods

### 2.1 Materials

Primary antibodies for glyceraldehyde 3-phosphate dehydrogenase (GAPDH), leukemia inhibitory factor (LIF), SH2 containing protein tyrosine phosphatase-2 (SHP2), and S100 calcium-binding protein β (S100β), heterogeneous nuclear ribonucleoprotein A1 (hnRNP), β-actin, and Protein A/G PLUS-Agarose were obtained from Santa Cruz Biotechnology (Santa Cruz, CA). Primary antibodies for phospho-tyrosine 705 STAT3 (p^Y705^-STAT3), acetyl-lysine 685 STAT3 (ac-STAT3), total STAT3, phospho-tyrosine 1007/1008 Janus kinase 2 (JAK2) (p^Y1007/1008^-JAK2), total JAK2 (JAK2), and ubiquitin were purchased from Cell Signaling Technologies (Danvers, MA). Primary antibodies for cardiotrophin-1 (CT-1) and glial fibrillary acidic protein (GFAP) were obtained from Abcam (Cambridge, MA). The primary antibody for protein-tyrosine phosphatase 1B (PTP1B) was from Millipore (Billerica, MA). Polyvinylidene difluoride (PVDF) membranes were obtained from Bio-Rad (Hercules, CA, USA). The ECL Plus Western blotting detection reagent was from Amersham Pharmacia Biotech Inc. (Piscataway, NJ). All other reagents were of the highest quality available and purchased from Sigma (St. Louis, MO).

### 2.2 Animals and animal care

All procedures were in agreement with standards for the care of laboratory animals as outlined in the NIH Guide for the Care and Use of Laboratory Animals. All procedures were administered under the auspices of the Animal Resource Services of the University of California, Davis, which is accredited by the American Association for the Accreditation of Laboratory Animal Care. Experimental protocols were approved before implementation by the University of California, Davis, Animal Use and Care Administrative Advisory Committee, and were administered through the Office of the Campus Veterinarian.

**Fig. 1:**
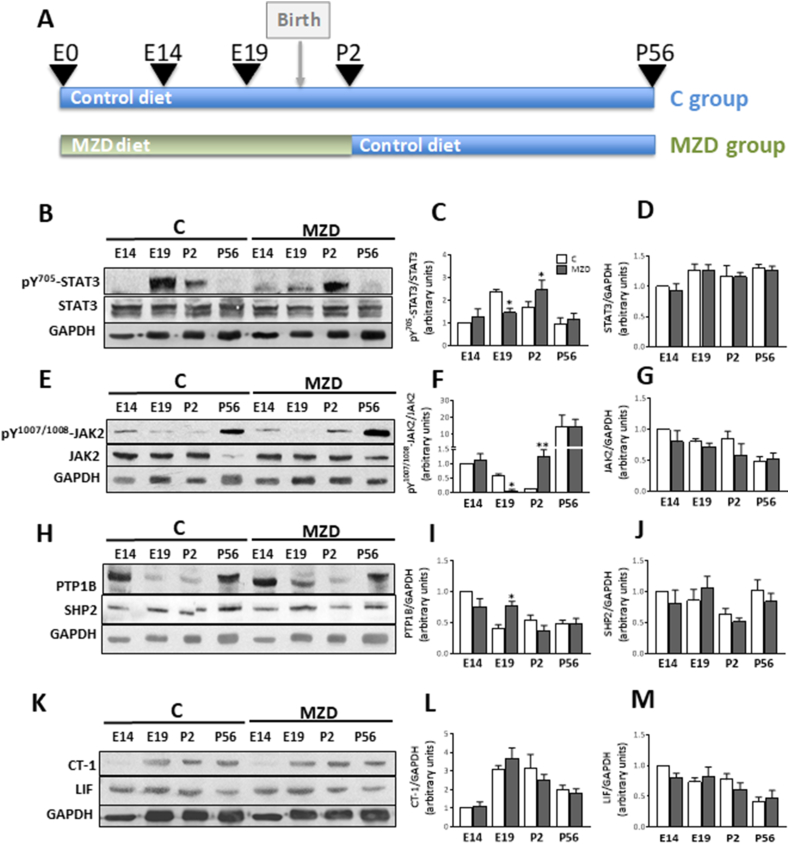
**Gestational MZD affects different steps in the STAT3 pathway in the offspring brain cortex at different developmental stages.** Dams were fed ad libitum either a control (C) or a marginal zinc diet (MZD) from gestation day 0 until E14, E19 and P2, at which time dams, and subsequently (after P20) the offspring were fed a control diet until P56. **A)** Experimental design. B-M) Brain cortex homogenates were prepared as described in the Materials and methods section. Western blots for **B-D**) phosphorylated STAT3 at tyrosine-705 (p^Y^^705^-STAT3), total STAT3, and GAPDH; **E-G**) phosphorylated JAK2 at tyrosine-1007/1008 (p^Y^^1007/1008^-JAK2), total JAK2, and GAPDH; **H-J**) PTP1B, SHP2 and GAPDH; and **K-M**) CT-1, LIF and GAPDH. GAPDH was used as loading control. After quantifications of bands, values were calculated as the ratios **C**) p^Y^^705^-STAT3/STAT3, **D**) STAT3/GAPDH, **F**) p^Y^^1007/1008^-JAK2/JAK2, **G**) JAK2/GAPDH, **I**) PTP1B/GAPDH, **J**) SHP2/GAPDH, **L**) CT-1//GAPDH and **M**) LIF/GAPDH. For all proteins values were normalized to those of the E14 control group. Results are shown as means ± S.E.M and are the average of 6 litters per group per developmental stage. *, *p* ≤ 0.05; **, *p* ≤ 0.01 are significantly different compared to the respective control at each developmental stage (Student's *t*-test).

The general experimental design is summarized in Fig. 1A. Adult Sprague-Dawley rats (Charles River, Wilmington, MA) (200–225 g) were housed individually in suspended stainless steel cages at a temperature (22–23 °C) and photoperiod (12-h light/dark)-controlled room. An egg white protein-based diet with adequate Zn content (25 μg Zn/g) was used as a control diet [19]. Animals were fed a control diet for one week before breeding. Males and females were caged together overnight and the following morning the presence of a sperm plug confirmed successful breeding. At E0, dams were randomly assigned to one of two experimental diets: control diet (25 μg Zn/g diet, C) or a diet containing a marginal Zn concentration (10 μg Zn/g diet, MZD) and fed these diets until E14 (n = 6), E19 (n = 10), or P2 (n = 6). Dam food intake was recorded daily, and body weight was measured at 5-day intervals. As from P2 and until weaning at P20, pups were culled to 8 per litter and all dams were fed a control diet. Pups were then separated by gender and fed the control diet until P56. We define as marginal zinc concentration as the dietary zinc content that will support a normal overall maternal and offspring outcome, without the typical alterations (maternal anorexia and teratogenesis) associated with overt zinc deficiency.

Dams at E14 and E19, and their offspring at P2 and P56 were anesthetized with isoflurane (2 mg/kg body weight). At E14 and E19, dams were submitted to laparotomies, the gravid uterus was removed, and fetuses were collected, weighed and examined for gross structural malformations. Fetal rat brains were removed, weighed and either immediately processed for immunofluorescence or kept on ice to dissect cortical tissue (CT)-enriched regions which were subsequently frozen in liquid nitrogen and stored at −80 °C. In E14 embryos, CT comprised the cortical neuroepithelium with the subventricular zone (SVZ) and ventricular zone (VZ); in E19 embryos, CT comprised the cortical plate with the SVZ and VZ. P2 and P56 offspring brain/brain cortices were dissected after rat euthanasia, and processed as described above.

### 2.3 Preparation of total, nuclear and cytosolic fractions

For preparation of total tissue extracts, CTs were manually homogenized (15 mg of tissue/100 μl of lysis buffer: 50 mmol/L Tris pH 7.5, 150 mM NaCl, 2 mM EDTA, 2 mM EGTA, 50 mM NaF, 2 mM NaVaO_4_ containing inhibitors of proteases and phosphatases and 1% (v/v) Igepal). Samples were incubated at 4 °C for 20 min and then centrifuged at 10,000×*g* for 20 min. Supernatants were stored at −80 °C. Protein concentration was determined immediately before starting the assay [20].

Nuclear and cytosolic fractions were isolated as previously described [21]. Briefly, CTs were manually homogenized in ice-cold hypotonic buffer (15 mg of tissue/100 μl of buffer A: 10 mM HEPES (pH 8.0), 1.5 mM MgCl_2_, 5 mM KCl, 0.5 mM dithiothreitol, 0.4 mM NaVaO_4_, 0.5 mM PMSF and inhibitors of proteases and phosphatases). An equal amount of buffer A containing 0.2% (v/v) of Igepal was added and samples were incubated on ice for 10 min. After 15 min of centrifugation at 850×*g*, the supernatant (cytosolic fraction) was separated and stored at −80 °C. The pellet was rinsed with buffer A and centrifuged twice at 850×*g* for 2 min. The nuclear pellet was gently resuspended in 100 μl of buffer B (20 mM Tris (pH 8.0), 25% (v/v) glycerol, 1.5 mM MgCl_2_, 0.4 M NaCl, 0.2 mM EDTA, 0.5 mM dithiothreitol, 0.4 mM NaVaO_4_, 0.5 mM PMSF and inhibitors of proteases and phosphatases) and incubated for 15 min on ice. After centrifuging for 30 min at 10,000×*g*, the supernatant (nuclear fraction) was transferred into new tubes and stored at −80 °C.

### 2.4 Western blot analysis

Cell extracts containing 20–35 μg protein were added with 4× Laemmli sample buffer and heated for 5 min at 95 °C. Proteins were separated by 8–12% (w/v) polyacrylamide gel electrophoresis and electroblotted onto PVDF membranes. Colored molecular weight (Bio-Rad, Hercules, CA) and biotinylated (Cell Signaling Technologies, Danvers, MA) standards were run simultaneously. Membranes were blocked for 1 h in 5% (w/v) nonfat milk and incubated at 4 °C overnight with the corresponding primary antibodies (1:1000–1:5000) in 5% (w/v) bovine albumin serum (BSA) in TBS-Tween. After incubation with the secondary antibodies (1:10,000–1:30,000), the conjugates were visualized using an ECL system in a Phosphoimager 840 (Amersham Pharmacia Biotech. Inc., Piscataway, NJ).

### 2.5 Immunofluorescence

Fetal rat brains were dissected out and fixed in a 4% (w/v) solution of paraformaldehyde in PBS overnight. Tissues were washed with PBS twice before cryoprotection by incubations in 15% (w/v) sucrose in PBS for 24 h, and subsequently in 30% (w/v) sucrose for 3 d. Brains were then submerged in Cryoplast (Biopack, Buenos Aires, Argentina), frozen, cut into 18-μm sections on a Leica CM 1850 cryotome (Leica Microsystems, Nussloch, Germany) and mounted on positively charged microscope slides. Sections were blocked in 1% (v/v) donkey serum in 0.1% (v/v) Triton X-100 in PBS for 45 min and then incubated overnight at 4 °C with GFAP (1:700) and S100β (1:100) primary antibodies. Sections were washed once in 0.1% (v/v) Triton X-100 in PBS and once in 0.1 M phosphate buffer, pH 7.4, and later incubated for 2 h at room temperature with the corresponding Cy2 or Cy3-conjugated secondary antibodies (1:1000) (Jackson Immuno Research Co. Laboratories West Grove, PA). Cell nuclei were stained with Hoechst 33342.

Sections were imaged using an Olympus FV 1000 laser scanning confocal microscope (Olympus, Japan) or an Olympus BX50 epifluorescence microscope equiped with a Cool-Snap digital camera. Image Pro software (Rockville, MD) was used to merge and analyze the resulting micrographs. GFAP-positive cells were counted using ImageJ (National Institutes of Health, Bethesda, MD, USA) and results were expressed as the number of positive cells per area. At least 300 cells were counted and analyzed in three independent experiments performed in triplicate on four animals per group. Colocalization of GFAP/S100β was analyzed by laser confocal microscopy, and optical sections (Z = 1 μm) of confocal epifluorescence images were obtained sequentially by means of a 60× (NA, 1.35; digital zoom of 2.2) oil objective.

### 2.6 Immunoprecipitation

Fetal brain cortices were lysed in 1% (v/v) Nonidet P-40 buffer (10 mM Tris-HCl (pH 7.4), 150 mM NaCl, 1 mM EDTA, 1% (v/v) Nonidet P-40, 5% (v/v) glycerol, and proteases and phosphatase inhibitor cocktails). Lysates were cleared by centrifugation at 13,000×*g* for 10 min. Protein concentration was determined immediately before starting the assay. Protein lysates (400 μg100 μl)were immuno precipitated using STAT3 or PTP1B antibodies(4 μg)and immune complexes were collected on protein A/G agarose beads. The pellets were washed twice with lysis buffer Proteins were added with 4 x Laemmli sample buffer and heated for 5 min at 95 °C before Western blots. After SDS-PAGE and protein transfers, membranes for the STAT3 immunoprecipitates were probed with primary antibodies for PTP1B, SHP2, STAT3, p^Y705^-STAT3, JAK2, β-actin and α-tubulin, while membranes for PTP1B immunoprecipitates were probed with primary antibodies for ubiquitin, JAK2 and PTP1B. After an overnight incubation at 4 °C, membranes were incubated with the corresponding HRP-conjugated secondary antibodies and visualized using chemiluminescence detection in a Phosphoimager 840 (Thermo Fisher Scientific, Waltham, MA).

### 2.7 PTP1B enzyme activity assay

PTP1B activity was measured as previously described with minor modifications [22,23]. Briefly, offspring CTs were lysed in 1% (v/v) Nonidet P-40 buffer (10 mM Tris-HCl (pH 7.4), 150 mM NaCl, 1 mM EDTA, 1% (v/v) Nonidet P-40, 5% (v/v) glycerol, and protease inhibitors). Total proteinlysates(400 μg protein) were immunoprecipitated with the PTP1B antibody (4 μg)using protein A/G PLUS-agarose beads. The precipitate was washed twice with ice-coldlysis buffer and then resuspended in 50μl of reaction buffer (50 mM HEPES (pH 5.0), 1 mM EDTA, 100 mM NaCl and 5 mM DTT). After addition of the substrate (50 μl of 2 mM p-nitrophenyl phosphate) samples were incubated at 25 °C for 5 min. Then, 100 μl of BIOMOL Green reagent (Enzo Life Sciences, Inc., Farmingdale, NY) were added and samples incubated at 25 °C for 10 min. The absorbance was measured at 640 nm using a Wallac Victor 2 plate reader (PerkinElmer Life Sciences, Waltham, MA).

### 2.8 Electrophoretic mobility shift assay (EMSA)

Oligonucleotides containing the consensus sequence for STAT3 (5′-GAT CCT TCT GGG AAT TCC TAG ATC-3′) were end-labeled with (^32^P)-ATP using T4 polynucleotide kinase and purified using Chroma Spin-10 columns. Samples were incubated with labeled oligonucleotide (20,000–30,000 rpm) for 20 min at room temperature in 1× binding buffer [5× binding buffer: 50 mM Tris-HCl buffer, pH 7.5, containing 20% (v/v) glycerol, 5 mM MgCl_2_, 2.5 mM EDTA, 2.5 mM DTT, 250 mM NaCl and 0.25 mg/ml poly(dI-dC). Products were separated by electrophoresis in a 6% (w/v) non-denaturing polyacrylamide gel using 0.5 × TBE (Tris/borate 45 mM, EDTA 1 mM) as the running buffer. Gels were dried and the radioactivity quantified in a Phosphoimager 840 (Amersham Pharmacia Biotech. Inc., Piscataway, NJ).

### 2.9 Statistical analysis

Student unpaired *t*-test was performed to compare between two groups by using the routines available in SAS (SAS Institute Inc., Cary, NC). A p-value < 0.05 was considered statistically significant. Values are given as means ± S.E.M. The litter was considered the statistical unit.

## 3 Results

### 3.1 Animal outcome

Maternal MZD did not affect maternal and offspring outcomes at E14, E19, and P2. However, maternal MZD led to a higher (8.2%, (p < 0.05) body weight of the female offspring at P56, and a trend (9.6%, p = 0.07) for higher body weight of the male offspring compared to controls (Table 1). The ratio brain/body weight at P56 was 26 (p < 0.05) and 9.4% (p < 0.06) lower in MZD females and males, respectively, than in controls. As previously published for this set of animals [4], zinc content in the MZD offspring brain was lower at E14, E19 and P2 compared to controls.

**Table 1:** Outcome parameters in dams and their offspring fed control or MZD diets.

Parameter	Control	MZD
**E14**		
Dam cumulative food Intake (g)	324 ± 19	324 ± 14
Maternal weight gain (g)	67.4 ± 8.7	82.2 ± 6.5
Fetal weight (mg)	166 ± 5	189 ± 20
Placenta weight (mg)	163 ± 7	184 ± 16
Live fetuses/litter	14.7 ± 1.0	13.2 ± 1.0
**E19**		
Dam cumulative food intake (g)	600 ± 40	569 ± 30
Maternal weight gain (g)	154 ± 8	146 ± 9
Fetal weight (g)	2.36 ± 0.06	2.42 ± 0.04
Placenta weight (mg)	490 ± 13	460 ± 16
Brain weight (mg)	93 ± 3	94 ± 2
Brain weight/fetal weight	0.039 ± 0.003	0.037 ± 0.001
Live fetuses/litter	15.0 ± 0.9	14.0 ± 0.7
**P2**		
Dam cumulative food intake (g)	618 ± 39	593 ± 50
Pup weight (g)	7.4 ± 0.7	7.7 ± 0.3
Brain weight (mg)	228 ± 9	247 ± 10
Brain weight/pup weight	0.032 ± 0.001	0.032 ± 0.001
**P56**		
Offspring cumulative food intake (P3–P56) (g)	796 ± 25	684 ± 31
Offspring outcome		
Male body weight (g)	364 ± 5	399 ± 17
Female body weight (g)	233 ± 4	252 ± 8*
Male brain weight (g)	1.515 ± 0.002	1.464 ± 0.003
Female brain weight (g)	1.46 ± 0.49	1.25 ± 0.23
Male brain weight/body weight (×1000)	4.16 ± 0.15	3.77 ± 0.03
Female brain weight/body weight (×1000)	6.31 ± 0.20	4.80 ± 0.17*

At E0, dams were fed ad libitum C or MZD diet until E14 (n = 6), E19 (n = 10), and P2 (n = 6), at which time the offspring was weaned to the control diet until P56 (n = 6). Dam cumulative food intake was recorded daily from E0 to E14, E19 and P2. After P2, food intake was recorded every 5 d for dams until weaning and for the offspring until P56. Values are shown as means ± S.E.M. *Significantly different from control (*p* < 0.05; one-way ANOVA).

### 3.2 Maternal MZD modulates offspring brain cortex STAT3 activation during the period of active astrogliogenesis

Our previous study showed that gestational MZD in rats causes a decrease in whole brain STAT3 tyrosine phosphorylation at E19 [18]. We now investigated whether STAT3 activation is altered by maternal MZD in specific brain regions and developmental stages. STAT3 activation was evaluated by measuring p^Y^^705^-STAT3 by Western blot in the offspring CT at E14, E19, P2 and P56. Maternal MZD differentially affected STAT3 tyrosine phosphorylation depending on the developmental stage. Maternal MZD did not affect CT p^Y^^705^-STAT3 levels at E14 and P56, while they were significantly lower (51%; p < 0.01) at E19 when compared to controls (Fig. 1B and C). In contrast, p^Y^^705^-STAT3 levels were higher (15%; p < 0.05) in MZD than control CT at P2 (Fig. 1B and C). Maternal MZD did not affect total STAT3 protein levels at all developmental stages (Fig. 1 B,D), suggesting that maternal MZD regulates the activation (tyrosine phosphorylation), but not the expression of STAT3 in the developing brain cortex.

We next investigated whether the observed alterations in STAT3 phosphorylation caused by maternal MZD were dependent on alterations in upstream and downstream events, i.e. activating cytokines (CT-1, LIF), kinases (JAK2) and phosphatases (PTP1B and SHP2). In agreement with the pattern of STAT3 activation, JAK2 phosphorylation levels at Tyr 1007/1008 (p^Y^^1007/1008^-JAK2) did not vary between MZD and control groups at E14 and P56, while at E19 and P2 JAK2 phosphorylation levels were 92% lower and approximately 10-fold higher, respectively, compared to controls (Fig. 1E and F). Total JAK2 levels were similar between groups at all developmental stages, suggesting that maternal MZD regulates JAK2 activation, but not JAK2 expression.

We next investigated whether STAT3 phosphatases, i.e. PTP1B and SHP2, could be the potential regulators of STAT3 phosphorylation levels in MZD brain cortex. Protein levels of PTP1B in the E19 CT were lower (51%; p < 0.05), while at all other developmental stages they were similar, in MZD compared to controls (Fig. 1H and I). SHP2 levels were similar between groups throughout development (Fig. 1H,J).

During astrogliogenesis, STAT3 is activated by extracellular signals such as CT-1 and LIF [16,17,24,25]. Thus, we next measured by Western blot the levels of cytokines CT-1 and LIF. CT-1 and LIF protein levels were similar in MZD and control CT at all the tested developmental stages (Fig. 1K–M).

### 3.3 Maternal MZD affects the interactions of STAT3 with JAK2, PTP1B and cytoskeleton proteins in the offspring cortical tissue

Data showed that the critical timing of initiation of cortical STAT3 activation is E19, which coincides with the beginning of active astrogliogenesis. At this time point, STAT3 was particularly susceptible to the deleterious effects of gestational MZD. Thus, this time was selected to further study potential molecules/mechanisms involved in maternal MZD-mediated impairment of fetal brain STAT3 activation.

**Fig. 2:**
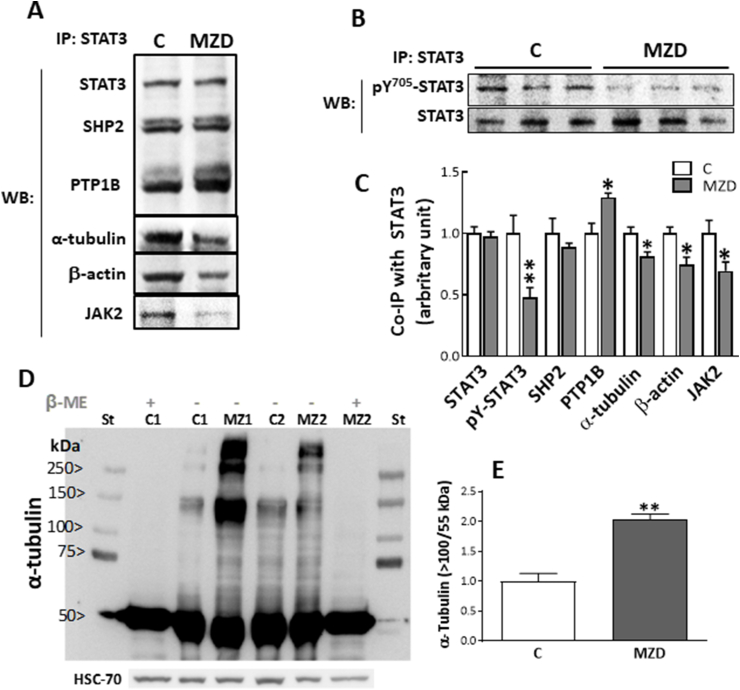
**Gestational MZD alters the interactions of STAT3 with the tyrosine phosphatase PTP1B and cytoskeleton proteins in the E19 brain CT. A-C**) STAT3 was immunoprecipitated from E19 CT tissue lysates as described in the Materials and methods section. A) Western blots for PTP1B, SHP2, α-tubulin, β-actin and STAT3 in the STAT3 immunoprecipitates (IP). **B**) Western blot for phosphotyrosine-705 STAT3 (p^Y^^705^-STAT3) and STAT3 I in the STAT3 immunoprecipitates (IP). **C**) After quantifications of bands, values were calculated as the ratios p^Y^^705^-STAT3/STAT3, SHP2/STAT3, PTP1B/STAT3, α-tubulin/STAT3, and β-actin/STAT3, and normalized to control levels. **D,E)** Brain CT was subjected to non-reducing homogenization and SDS-PAGE. **D**) Upper panel: Western blots for α-tubulin in control (C1,C2) and MZD (MZ1, MZ2) samples treated without (−) or with (+) addition of 1% (v/v) β-mercaptoethanol. St: molecular weight standard. HSC70 was assessed as loading control. **E)** After quantification, the ratio of intensity between bands of molecular weight higher than 100 kDa/50 kDa was calculated. Results are shown as means ± S.E.M. of six litters per group. *, *p* ≤ 0.05; **, *p* ≤ 0.01 are significantly different compared to controls (Student's *t*-test).

To examine whether zinc deficiency could affect the interactions of STAT3 with JAK2 and the phosphatases PTP1B and SHP2, we immunoprecipitated STAT3 from E19 CT and did immunoblotting for the interacting proteins. Compared to controls, maternal MZD caused a decrease (31%; p < 0.05) in the amount of JAK2 that co-immunoprecipitated with STAT3 (Fig. 2 A,C). In contrast, higher amounts (29%; p < 0.05) of PTP1B co-immunoprecipitated with STAT3 in MZD CT compared to controls, while those of SHP2 were similar between groups (Fig. 2 A,C). As expected, immunoprecipitated STAT3 showed lower tyrosine phosphorylation (52%; p < 0.05) and similar total STAT3 levels in E19 MZD CT than in controls (Fig. 2 B, C).

In terms of cytoskeletal proteins, the amount α-tubulin and β-actin that co-immunoprecipitated with STAT3 was 19% (p < 0.01) and 26% (p < 0.05) lower, respectively, in the E19 CT from the MZD group compared to controls (Fig. 2D and E). Western blots of total CT proteins were subsequently ran in non-reducing conditions to evaluate if the decreased interactions STAT3-tubulin could be secondary to tubulin oxidation (Fig. 2D). The amount of tubulin aggregates (bands of molecular weight equal and higher than 100 kDa) relative to that of tubulin monomers was 103% higher (p < 0.003) in MZD E19 CT compared to controls (Fig. 2E). Tubulin oligomers disappeared when samples were treated with 1% (v/v) of the disulfide reductant β-mercaptoethanol (Fig. 2D). These results are in line with previous observations that, under conditions of Zn deficiency, tubulin aggregates are formed as a consequence of disulfide bond crosslinking [26].

### 3.4 Maternal MZD impairs PTP1B degradation via the ubiquitin pathway in E19 cortical tissue

**Fig. 3:**
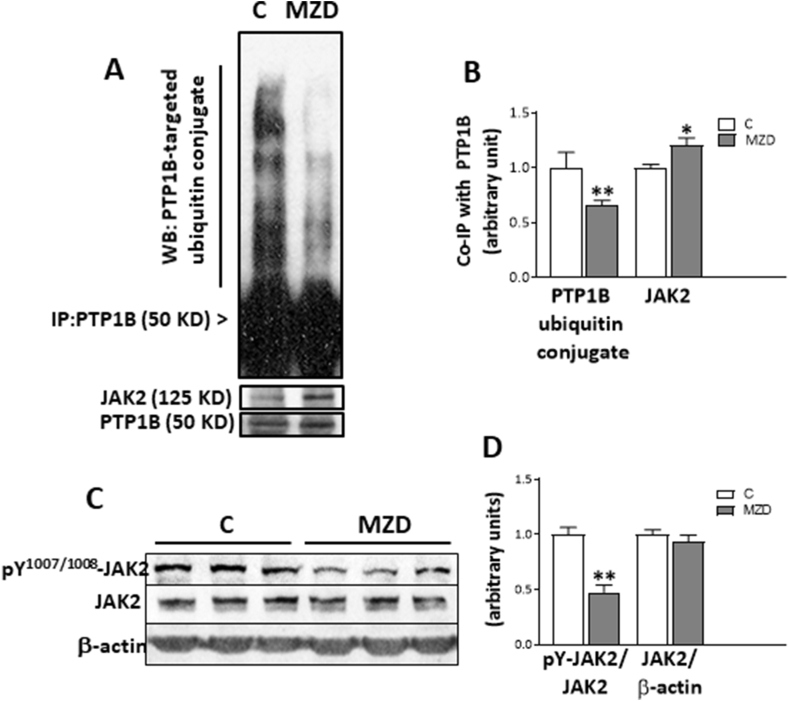
**Gestational MZD impaired PTP1B degradation via the ubiquitin/proteasome pathway and increased PTP1B-JAK2 interaction in E19 brain CT**. PTP1B was immunoprecipitated from E19 CT tissue lysates as described in the Materials and methods section. **A**) Western blots for ubiquitin conjugates, JAK2 and PTP1B in the PTP1B immunoprecipitates (IP). **B**) After quantifications of bands, values were calculated as the ratios ubiquitin conjugates/PTP1B and JAK2/PTP1B, and normalized to control levels. **C**) Western blot of phosphotyrosine-1007/008 JAK2 (p^Y^^1007/1008^ JAK2), total JAK2 (JAK2), and β-actin in lysates of E19 CT. **D**) After quantifications of bands, values were calculated as the ratios p^Y^^1007/1008^ JAK2/JAK2 and JAK2/β-actin, and normalized to control levels. Results are shown as means ± S.E.M. of six litters per group. *, *p* ≤ 0.05; **, *p* ≤ 0.01 are significantly different compared to controls (Student's *t*-test).

We subsequently focused on PTP1B given that STAT3 and JAK2 are substrates of this enzyme, and that the amount of PTP1B co-immunoprecipitated with STAT3 was affected by developmental MZD. During astrocyte precursor differentiation, PTP1B down-regulation occurs via ubiquitin/proteasome-mediated degradation [27]. Thus, we next examined whether maternal MZD could disrupt PTP1B ubiquitination. After immunoprecipitating PTP1B from E19 CT homogenates, we observed lower levels (34%, p < 0.01) of PTP1B-ubiquitin conjugates in E19 MZD CT than in controls (Fig. 3A and B). The amount of JAK2 co-immunoprecipitating with PTP1B was higher (20%; p < 0.05) in E19 MZD CT than in controls (Fig. 3 A, B). Accordingly, phosphorylation at tyrosine 1007 and 1008 of the co-immunoprecipitated JAK2 was 52% lower in MZD compared to control E19 CT (Fig. 3C and D). Taken together, results support the concept that an impairment of PTP1B degradation could be one of the mechanisms underlying zinc deficiency-mediated deactivation of the JAK2/STAT3 signaling pathway during early brain development.

### 3.5 Maternal MZD differentially affects cortical PTP1B activity at E19 and P2

**Fig. 4:**
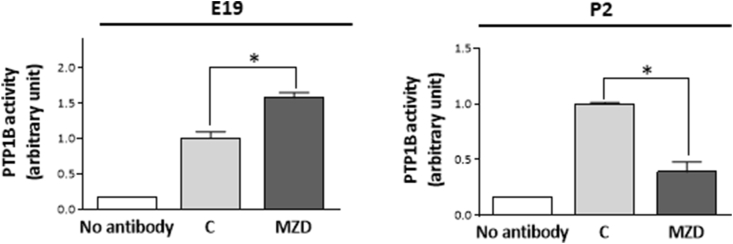
**Gestational MZD altered PTP1B enzymatic activity in the E19 and P2 CT.** PTP1B enzymatic activity for PTP1B was measured in A) E19 and B) P2 CT as described in the Materials and methods section. PTP1B activity was referred to PTP1B protein levels in the immunoprecipitate and normalized to control values. No antibody: the immunoprecipitation was done in the absence of the PTP1B primary antibody. Results are shown as means ± S.E.M. of six litters per group. * Significantly different compared to the respective control (*p* ≤ 0.05, Student's *t*-test).

Based on the reciprocal activation of STAT3 and JAK2 observed in E19 and P2 CT, we investigated if PTP1B varied accordingly. While PTP1B protein levels in CT at P2 was not significantly different, the enzymatic activity of PTP1B in MZD brain cortices was higher (57%; p < 0.05) at E19 (Fig. 4 A) and lower (60%; p < 0.05) at P2 (Fig. 4 B) than in controls at the corresponding developmental stage.

### 3.6 Maternal MZD impairs cortical nuclear translocation and acetylation of STAT3 at E19 and P2

**Fig. 5:**
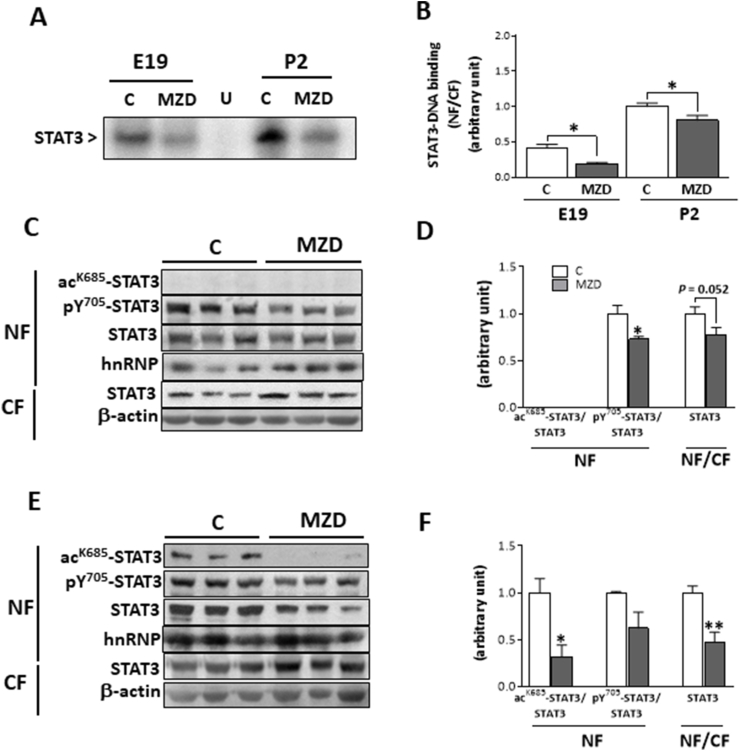
**Gestational MZD impaired STAT3-DNA binding and STAT3 acetylation in nuclear fractions form E19 and P2 brain CT.** Nuclear and cytosolic fractions were isolated from E19 and P2 brain CT as described in the Materials and methods section. **A,B)** EMSA for STAT3 in nuclear (NF) and cytosolic fractions (CF) isolated from E19 and P2 CT. To determine the specificity of STAT3-DNA complex, a control NF was incubated in the presence of a 100-fold molar excess of unlabeled oligonucleotide containing the consensus sequence for an unspecific (U) transcription factor before the binding assay. **A)** Representative image for NF, **B**) after the EMSA assay, bands were quantified and the ratio nuclear/cytosolic DNA binding (NF/CF) was calculated. Results were referred to the control P2 value (1**). C–F)** Western blots for ac^K685^-STAT3, p^Y^^705^-STAT3, STAT3 and hnRNP in NF; and STAT3 and β-actin in CF from **C,D**) E19 and **E,F)** P2 offspring CT. **C,E** show representative images. **D,F**) After quantifications of bands, values were calculated as the ratios p^Y^^705^-STAT3/STAT3 and ac^K685^-STAT3/STAT3 in NF and the ratio of STAT3 NF/STAT3 in CF and normalized to control levels. Results are shown as means ± S.E.M. of six litters per group. *, *p* ≤ 0.05; **, *p* ≤ 0.01 are significantly different compared to the respective control (Student's *t*-test).

STAT3 transactivating activity requires STAT3 tyrosine phosphorylation, and its subsequent dimerization and transport into the nucleus. We next examined the effects of developmental MZD on STAT3-DNA binding activity in nuclear fractions. At E19 and P2, the ratio of nuclear/cytosolic STAT3-DNA binding was significantly lower (54% and 19%, respectively, p < 0.05) in MZD CT compared to controls (Fig. 5A and B).

The deleterious effects of zinc deficiency on cytoskeleton assembly could affect the transport of active STAT3 from the cytosol into the nucleus. As measured by Western blot, the total STAT3 protein nuclear/cytosolic ratio was lower at E19 and P2 in MZD CT compared to controls (Fig. 5C–F). The ratio p^Y^^705^-STAT3/total STAT3 in MZD CT was lower at E19 (27%; p < 0.05) (Fig. 5C and D) but not at P2 compared to controls at the corresponding developmental ages (Fig. 5E and F).

STAT3 acetylation in the nucleus is another relevant mechanism leading to STAT3 activation. Acetylated (lysine-685) STAT3 levels in nuclear fractions were not detectable in both control and MZD CT at E19 (Fig. 5C and D). At P2, acetylation of nuclear STAT3 was 68% lower (p < 0.05) in the MZD offspring CT than in controls (Fig. 5E and F). Thus, impaired acetylation could contribute to the observed MZD-mediated disruption of STAT3 activation in the developing brain.

### 3.7 Maternal MZD affects cortical astrogliogenesis in the offspring

**Fig. 6:**
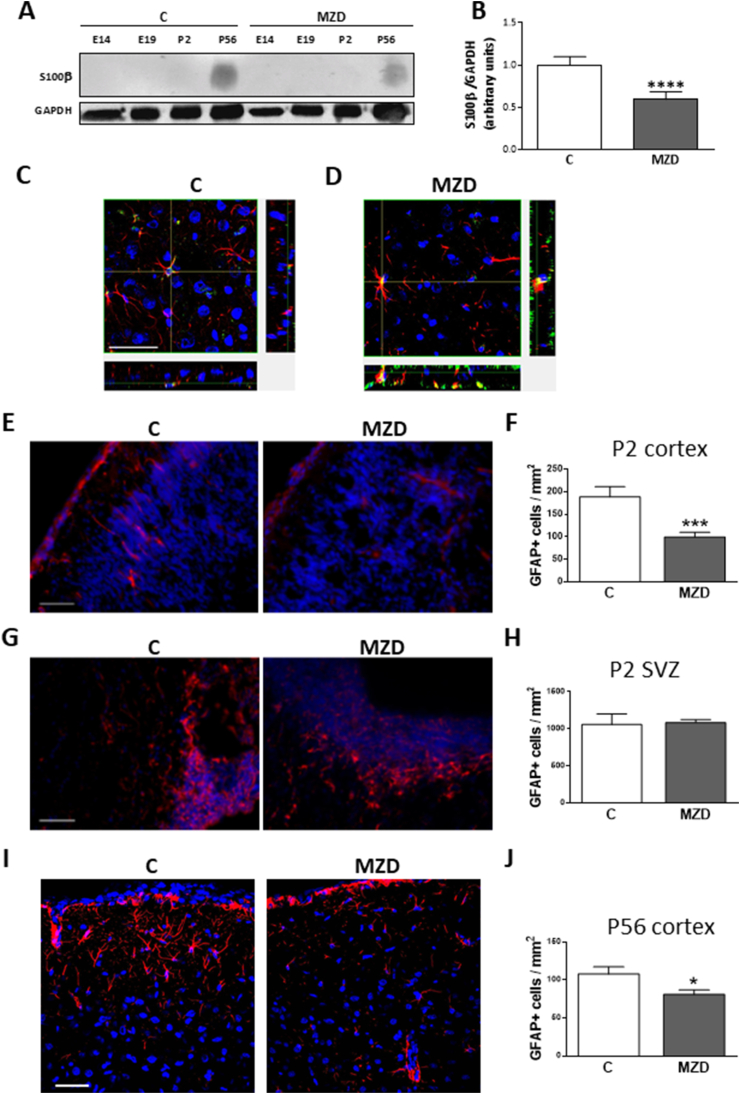
**Early developmental exposure to marginal Zn nutrition affects cortical astrogliogenesis in the offspring.** Dams were fed ad libitum either a control (C) or a marginal zinc diet (MZD) from gestation day 0 until E14, E19 and P2, at which time dams, and subsequently (after P20) the offspring, were fed a control diet until P56. **A)** Western blot for S100β and GAPDH. **B)** After band quantification, values were calculated as the ratio S100β/GAPDH and normalized to those of the control group. Results are shown as the mean ± S.E.M. of P56 brain cortices from 3 rats/group. ****, *p* < 0.0001 are significantly different from the control group (Student's t-test). **C,D**) Orthogonal reconstructions of GFAP (red fluorescence) and S100β (green fluorescence) single confocal optical sections in the z-axis at the level indicated by the yellow lines in P56 cerebral cortex from the **C)** control and **D)** MZD offspring. Scale bar 50 μm. **E,G,I**) Representative images of GFAP (red fluorescence) positive astrocytes measured by immunofluorescence in: **E)** the cerebral cortex, **G)** the SVZ at P2 and **I)** the cerebral cortex at P56 of C and MZD offspring. **F**,**H**, **J)** Results are expressed as GFAP positive cells/mm^2^ and shown as the mean ± S.E.M. of brain cortices from 4 litters/group, *, p < 0.05; ***, p < 0.001 are significantly different from the control group (Student's t-test). Scale bar 50 μm. (For interpretation of the references to colour in this figure legend, the reader is referred to the Web version of this article.)

We next investigated whether the developmental impairment of the STAT3 signaling pathway caused by zinc deficiency could affect the number of astrocytes in the offspring brain cortex. The astrocyte marker S100β was assessed by Western blot in the offspring CT at different developmental stages. S100β, a marker for mature astrocytes, was only detectable in the P56 brain cortex, and its levels were significantly lower (40%; p < 0.0001) in the brain cortex of the MZD offspring than in controls (Fig. 6A and B). As expected, S100β co-localized with GFAP in control and MZD P56 offspring brain cortex as evaluated by orthogonal reconstruction of confocal microscopy sections in the z-axis, (Fig. 6C and D). The effects of gestational and early postnatal marginal Zn deficiency on the astrocyte population at P2 and P56 was next evaluated by immunofluorescence using GFAP as an astrocyte marker. The number of astrocytes per area in the brain cortex was significantly lower in the brain cortex of MZD offspring at P2 (Fig. 6E and F) and P56 (Fig. 6I and J). The number of GFAP-positive cells per area in the SVZ at P2 was similar in both groups (Fig. 6G and H). This is in agreement with our previous results showing no significant differences between control and MZD offspring for Sox2 and Sox2/Ki67-positive NSC proportion in the SVZ, suggesting that MZD does not affect the population of proliferating NSCs present in the P2 SVZ [4]. The above findings support the concept that moderate zinc deficiency during pre- and early post-natal stages can negatively affect astrocyte generation, which can extend into adulthood. The above data suggest that maternal MZD can adversely and irreversibly affect astrogliogenesis and the total number of astrocytes in the young adult offspring brain cortex.

## 4 Discussion

This study shows that a decreased Zn availability during gestation and early postnatal stages affects cortical astrogliogenesis, in part as a consequence of a dysregulation of STAT3. The adverse effects of early developmental Zn deficiency on astrogliogenesis caused irreversible alterations in the astroglial population in the young adult rat brain. Maternal Zn deficiency adversely affects several steps in the JAK/STAT pathway, i.e. JAK2 and STA3 activation (phosphorylation), PTP1B degradation, STAT3-cytoskeleton interactions, and STAT3 acetylation.

Mild maternal Zn restriction in rats does not affect general maternal and fetal outcomes [18,[28], [29], [30], [31]]. However, it causes major changes in Zn homeostasis, NSC proliferation and neurogenesis [4,5], and affects E19 brain signaling [18,32,33]. Observations of impaired E19 brain STAT3 activation [18] and of a lower NSC number, suggested that astrogliogenesis could be also affected by gestational marginal Zn deficiency.

During brain development, STAT3 expression is region-specific [[34], [35], [36], [37]]. In the rat cortical tissue, the onset of STAT3 protein expression is evident at E14, being sustained throughout adulthood [37]. Accordingly, we observed that STAT3 protein levels were similar from E14 through P56 in both the control and Zn deficient offspring CT. STAT3 tyrosine phosphorylation varies with the developmental stage and its distribution depends on different stimuli [16,38]. In the Zn deficient CT, STAT3 tyrosine phosphorylation was lower at E19, higher at P2, and similar to control values at E14 and P56. A similar pattern was observed for the activation of the STAT3 tyrosine kinase, JAK2 [37,39]. This temporal dysregulation of JAK2/STAT3 activation by maternal Zn occurs during the astrogliogenesis-specific developmental period in rats, which further supports a link between STAT3 and Zn in the process of astrogliogenesis.

Maternal marginal Zn deficiency increased the protein levels and activity of the STAT3 phosphatase PTP1B in the E19 CT. Given that PTP1B dephosphorylates both JAK2 and STAT3 [[40], [41], [42]], this phosphatase emerges as a potential target for the adverse consequences of Zn deficiency on astrogliogenesis. In fact, indirect findings showed a possible connection between PTP1B and alcohol-associated Zn deficiency during pregnancy [43]. While prenatal alcohol exposure induces Zn deficiency and impairs Zn utilization by the offspring [[44], [45], [46]], PTP1B activity increased in the brain of rats exposed to alcohol during gestation [47]. While high protein levels and activity of PTP1B were observed in the marginal Zn deficient E19 CT, low activity and similar PTP1B protein levels occurred at P2. PTP1B can be responsive to cellular Zn status given that its activity is regulated by the cell redox status and by Zn *per se* [3,48]. Thus, Zn deficiency-associated increase in oxidant production can inhibit PTP1B activity, while Zn directly inhibits PTP1B at nanomolar concentrations [49,50]. The increased PTP1B protein levels found in the Zn deficient CT can be due to the observed decrease in PTP1B ubiquitylation, which would impair PTP1B degradation. While there is a lack of information about the effects of Zn on protein ubiquitylation, a potential target could be the E3 ubiquitin ligases. In fact, the structure of these enzymes is stabilized through eight conserved zinc-chelating residues that bind two Zn atoms and contains a core residue cysteine (Cys 3) [51]. Overall, the low levels of STAT3 and JAK2 activation in the Zn deficient E19 CT can be secondary to PTP1B activation, caused by a decrease in cellular Zn, or to increased PTP1B protein levels, caused by an impaired proteasomal degradation of the enzyme. Commonalities in STAT3, JAK2 and PTP1B activity/protein levels suggest that PTP1B may be a critical modulator of the JAK2/STAT3 signaling pathway.

Zn deficiency causes oxidative stress and oxidative damage to lipids, proteins and DNA [30], being tubulin a key target. Tubulin thiol oxidation as a consequence of Zn deficiency impairs tubulin polymerization both *in vitro* and *in vivo* [26]. One consequence of these alterations is a decreased nuclear translocation of several transcription factors, including NF-κB, NFAT, STAT1, STAT3 and Nrf2 [18,21,26,33,52,53]. Accordingly, Zn deficiency decreased STAT3 nuclear content in the E19 and P2 CT. In Zn deficient neuronal cells, the antioxidant α-lipoic acid reduced tubulin disulfides and restored polymerization [26] and STAT3 nuclear transport [18]. We did not observe β-actin polymers in the MZD CT (data not shown). However, actin and tubulin cross-talk is very important in the overall function of the cytoskeleton [54]. Thus, zinc deficiency-associated tubulin oxidation and depolymerization could affect the dynamics of the actin network and explain the observed decreased interaction of actin with STAT3 in the MZD brain cortex. While other mechanisms of direct regulation of STAT3 signaling by microtubules cannot be ruled out [55,56], the observed decreased STAT3-tubulin interaction and accumulation of tubulin oxidized oligomers in the Zn deficient E19 CT provide further evidence of tubulin oxidation as a major factor in STAT3 impaired nuclear translocation. Additionally, the observed low levels of nuclear STAT3 acetylation at lysine-685 in Zn deficient P2 brain cortex can also contribute to a decrease in nuclear STAT3. Thus, STAT3 acetylation is involved in STAT3 dimerization, DNA binding, transactivation activity, and nuclear import [57,58]. Taken together, both disruptions of STAT3 interactions with the cytoskeleton and decreased STAT3 acetylation can underlie the decreased offspring brain STAT3 nuclear translocation and DNA binding caused by developmental Zn deficiency.

In association with STAT3 dysregulation, a marginal zinc nutrition during gestation and until P2 impaired astrogliogenesis in the offspring brain, which extended into young adulthood. In mammals, astrogliogenesis is considered to be completed soon after birth and could be reactivated only in pathological situations. The astroglial population increases 6-8-fold in normal rats and mice during the first 3 postnatal weeks [59,60], being a local symmetric division of differentiated astrocytes the most important astroglial source in the postnatal cortex [12]. During development, astrocytes are generated from NSC only after these cells have generated neurons. While several signaling pathways are involved in astrogliogenesis, the JAK/STAT signaling cascade is particularly critical in this process [61] and different factors that stimulate astrogliogenesis, e.g. BMPs, basic fibroblast growth factor (bFGF), and Notch, need pre-activation of the JAK/STAT pathway [62,63]. Marginal gestational zinc deficiency alters neuronal specification in the mature brain [4]. This hints at a dysregulation of differentiation and migration of precursor cells during brain development which, in turn, could also affect the population of astrocytes in the adult cerebral cortex through alterations in the JAK/STAT signaling. On the other hand, we did not observe changes in GFAP-positive cells in the SVZ of P2 MZD offspring. This is in agreement with our previous results showing no significant differences in the proportion of Sox2 and Sox2/Ki67-positive NSCs for both control and MZD, which suggests that MZD does not affect the proliferating NSCs present in the P2 SVZ. These results are not discrepant with the observed reduction in the astroglial population in the brain cortex of P56 MZD offspring, as the production of new astrocytes from NSCs present in the SVZ is absent in the normal adult cortex [64]. Thus, the observed low number of GFAP-positive cells in the MZD offspring brain cortex at P2 is due to Zn deficiency-mediated STAT3 deregulation which impairs cell fate decisions to differentiate into astrocytes in the VZ and SVZ. As a consequence, the number of astrocytes that reaches the brain cortex and that can proliferate to generate more astrocytes in the mature brain is lower. This can in part explain the persistence of a low number of astrocytes in the P56 brain even after the postweaning consumption of Zn sufficient diets in the offspring exposed to early developmental zinc deficiency.

In summary, our results show that marginal dietary Zn deficiency during pregnancy and until P2 causes a disruption in astrogliogenesis which extends into adulthood even after reinstatement of a diet with adequate Zn concentration shortly after birth. Given the mechanisms underlying astrocyte generation in the rodent CNS [12], it may be speculated that, in hampering NSC proliferation at embryonic stages, Zn deficiency affects post-natal astrogliogenic sources and thus restricts the generation of both SVZ-progenitor-derived and local-division-derived astrocytes in the young adult brain. Dysregulation of STAT3, one of the important signaling pathway orchestrating astrogliogenesis, may be one of the main underlying mechanisms. STAT3 regulation by Zn deficiency occurs as a consequence of: i) PTP1B activation/overexpression leading to decreased JAK2 and STAT3 phosphorylation/activation; ii) tubulin oxidation, associated decreased interactions with STAT3 and consequent impaired nuclear translocation and (iii) decreased nuclear STAT3 acetylation. Alterations in astrocyte generation during development may contribute to dysfunctions in synaptic plasticity, neuropsychological disorders and brain tumors [65,66]. Results underscore the relevance of zinc during early brain development for normal astrogliogenesis and the potential adverse impact of its deficiency for the long-term and irreversible effects on brain structure and function, and risk for neurological diseases.

## Declaration of competing interest

The authors have no conflict of interest to declare.
